# The global burden of high fasting plasma glucose associated with zinc deficiency: Results of a systematic review and meta-analysis

**DOI:** 10.1371/journal.pgph.0001353

**Published:** 2023-03-13

**Authors:** James P. Wirth, Wu Zeng, Nicolai Petry, Fabian Rohner, Scott Glenn, William E. S. Donkor, Rita Wegmüller, Erick Boy, Keith Lividini

**Affiliations:** 1 GroundWork, Fläsch, Switzerland; 2 School of Health, Georgetown University, Washington, DC, United States of America; 3 Institute for Health Metrics and Evaluation, Seattle, WA, United States of America; 4 Research Delivery & Impact Division/International Food Policy Research Institute (IFPRI), Washington, DC, United States of America; University of South Carolina Arnold School of Public Health, UNITED STATES

## Abstract

Non-communicable diseases (NCDs) account for the largest share of the global disease burden, and increasing evidence shows that zinc deficiency (ZD) contributes to NCDs by inducing oxidative stress, insulin resistance, and impaired lipid metabolism. A systematic review and meta-analysis was conducted to determine whether ZD was associated with fasting plasma glucose (FPG), a key risk factor for NCDs. A random effects meta-analysis was conducted to determine the strength of the association in the form of an odds ratio (OR) and subsequently the population attributable risk (PAR) with population prevalences of high FPG. The disease burden from high FPG attributable to ZD was expressed as disability adjusted life years (DALYS). Data from seven studies were obtained as part of the systematic review. The meta-analysis shows a significant (p<0.01) inverse relationship between ZD and high FPG (OR = 2.34; 95% CI: 1.16, 4.72). Globally, the PAR of ZD’s contribution to high FPG is 6.7%, with approximately 8.2 million high FPG DALYs attributable to ZD. Cardiovascular diseases, diabetes, and chronic kidney diseases account for more than 90% of the total DALYs. Total DALYs attributable to ZD are largest in the “Southeast Asia, East Asia, and Oceania” and “High Income” Super Regions. While the disease burden is highest among populous countries (e.g., China, India, USA), the population-standardized DALYs are highest among island nations, particularly island nations in the South Pacific and Caribbean. While ZD accounts for a small share of the high FPG disease burden, the total number of DALYs far surpasses other estimates of the disease burden attributable to ZD, which focus on diarrheal diseases in childhood. Zinc interventions are urgently needed to help address the increasing disease burden from NCDs, and the double burden of malnutrition.

## Introduction

Non-communicable diseases (NCD; e.g., cardiovascular diseases, diabetes, cancers, chronic respiratory diseases) are a leading contributor to the burden of disease globally, and account for a majority of the global deaths [1]. The burden of disease per capita related to NCD is highest in high-income countries, but is rising in low- and middle-income countries [2]. A key metric of disease burden is the Disability-Adjusted Life Years (DALY), which standardizes the loss of life from death and disability and enables a direct comparison of disparate disease states. Globally, DALYs from NCDs have almost doubled between 1990 and 2017 [3], and most countries are not on track to meet the 2030 Sustainable Development Goals’ targets [1].

Obesity, hypertension, and insulin resistance are well-documented risk factors for NCDs, and there is increasing evidence that low circulating zinc levels contribute to NCDs by inducing oxidative stress [4], insulin resistance [5], and by altering lipid metabolism [6]. Multiple randomized controlled trials (RCTs) have investigated the effect of zinc supplementation on risk factors for diabetes and cardiovascular disease such as total cholesterol, low density lipoprotein (LDL) cholesterol, high density lipoprotein (HDL) cholesterol, and triglycerides, or direct measures of the disease such as fasting plasma glucose (FPG) or glycated hemoglobin (HbA1c) [5,7–11]. Several meta-analyses have systematically analyzed the results of these RCTs [5,7,9,10]. Although the inclusion criteria of the different meta-analyses varied, almost all analyses revealed that zinc administration reduced FPG, HbA1c, and insulin resistance. Pompano & Boy [7] included RCTs that administered zinc supplements at doses below 100mg/ day to healthy and unhealthy patients, subgrouping the interventions by dose and duration. They found larger effects for almost all indicators when the zinc dose administered was <25mg/d and for trials with a duration of 12 weeks or more. Similar results have been reported from a meta-analysis by Capdor *et al*. [10], which found a significant reduction in FPG in the intervention groups receiving zinc supplements. In sub-group analyses by health status, the effect was stronger in the diabetic sub-group and ceased to exist in the healthy subgroup [10]. In contrast, Wang *et al*. [5] found no difference in the magnitude of the effect between trials using different doses or duration.

These studies suggest that zinc supplementation has a therapeutic effect among diabetic patients and may delay the progression of zinc-associated chronic diseases. Specifically, there is mounting evidence that poor zinc status can increase levels of FPG, a key contributor of type-2 diabetes, cardiovascular diseases, and certain cancers [12], and that increasing dietary zinc could reduce the risk of developing type-2 diabetes [13] and the risk factors of certain cardiovascular diseases [14]. However, the strength of the association between zinc supplementation and elevated FPG has not yet been fully quantified among individuals who are zinc deficient compared with zinc-replete individuals.

Historically, zinc deficiency (ZD) and its contribution to the burden of disease has been linked to under-nutrition related conditions in children, such as diarrhea [15], pneumonia [16], and growth faltering [17]; and the disease burden from ZD has been quantified on the same basis for more than a decade [18,19] for both characterization and cost-effectiveness analysis of zinc interventions [20,21]. NCDs have not been included in these analyses; however, due to the growing body of evidence showing ZD’s association with insulin resistance and type-2 diabetes, quantification of the zinc-related disease burden from NCD in all population groups is warranted.

The objective of this study was to estimate the global disease burden of NCDs attributable to ZD. Our study had four aims, which were all achieved. The first aim was to conduct a systematic review of studies that determined elevated FPG among those with and without ZD. The second aim was to characterize the odds ratio (OR) for elevated FPG from ZD. The third aim was to determine the population attributable risk (PAR) for all countries using the OR and the estimated prevalence of elevated FPG for each country. The final aim was to estimate the total number of DALYs from elevated FPG attributable to ZD using each country’s calculated PAR and its estimated burden of disease due to elevated FPG determined from the Global Burden of Disease (GBD) project.

By estimating the OR based on a systematic review of the literature, this study makes an empirical contribution to literature examining the association between the ZD and high FPG. Furthermore, this analysis makes a methodological contribution by utilizing the results from the meta-analysis and data from the GBD project to estimate the disease burden from high FPG that is associated with ZD.

## Methods

### Experimental design

**Fig 1:**
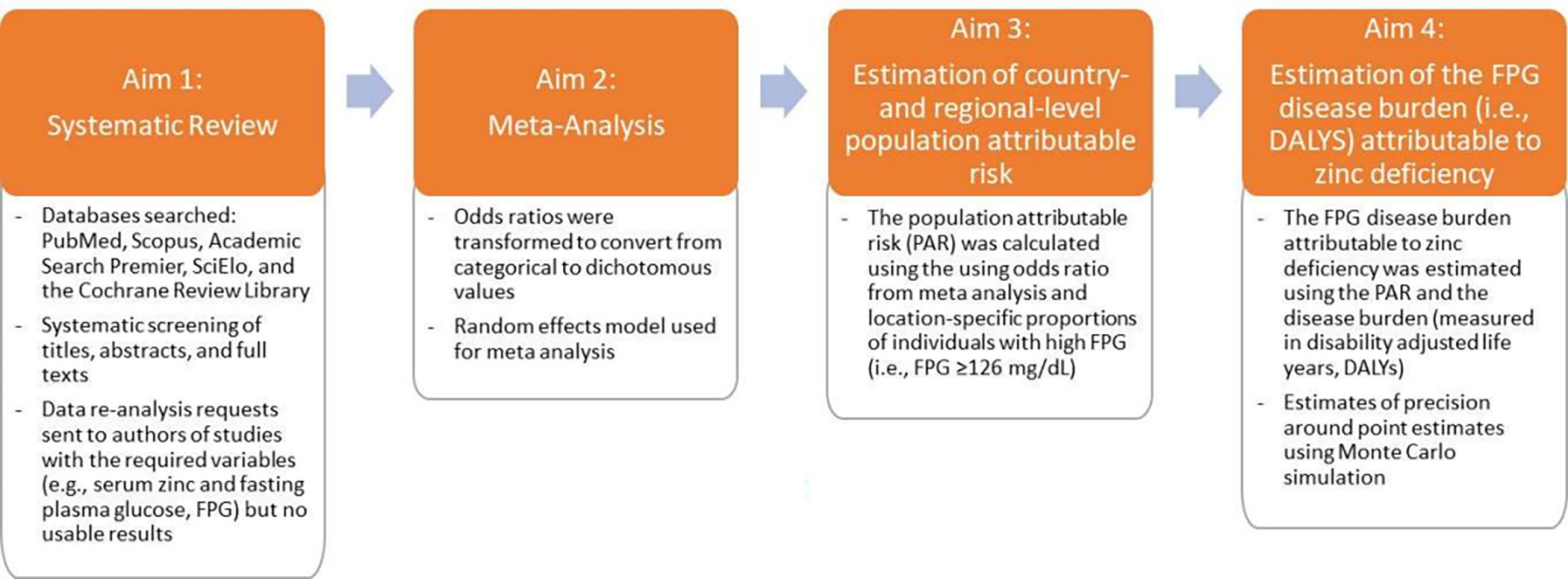
Analytical phases of the research study.

The aims and experimental design of this study are shown in **Fig 1**. The study commenced with a systematic review of literature databases (Aim 1) to identify studies from multiple population groups from several countries and locations that specifically included information on both zinc status (deficient vs. sufficient) and FPG status (elevated vs. normal). The systematic review also included an author outreach step, whereby authors of studies that contained zinc status and FPG indicators were contacted and requested to re-analyze their data if the article did not already present the results in a usable format. Subsequently, a random effects meta-analysis was conducted to determine the strength of the association in the form of an OR (Aim 2) that could be used for the estimation of the PAR of ZD inducing high FPG (Aim 3). Consequently, the *disease burden attributable to ZD* was calculated as the product of the PAR and the disease burden from high FPG—presented in DALYs and presented separately for each condition (Aim 4). A detailed description of the procedures undertaken for the meta-analysis and estimation of the PARs and disease burden attributable to ZD are presented in the Statistical Analysis section below.

At the outset of the study, the research team also conducted a systematic review and meta-analysis of the dichotomous relationship between ZD and high LDL cholesterol. This meta-analysis yielded few studies and did not show an association between the two factors. As such, the meta-analysis results were not used to estimate the proportion of the disease burden from high LDL cholesterol that was attributable to ZD (see Fig A and Fig B in S1 Text for a summary of these findings).

### Systematic review

#### Search strategy

A literature search was conducted in PUBMED, Scopus, SciElo, and Academic Search Premier databases, and the Cochrane Review Library (last search in all databases November 2021) to identify articles that examined the association between zinc status and fasting blood glucose. The search term used was as follows: (zinc OR zinc* OR zn) AND ("deficiency" OR "status" OR “level” OR “intake” OR “insufficiency”) AND ("fasting glycemia" OR "fasting glucose" OR "glucose" OR "glycemic" OR "glycemia" OR “fasting blood glucose” OR “blood glucose”) AND (epidemiology* OR "epidemiological studies" OR "case control" OR “case-control” OR “retrospective study” OR "cohort study" OR “study” OR “trial”). Prior to screening, any duplicates were removed and only studies written in English, Spanish, or Portuguese were retained.

#### Inclusion and exclusion criteria of title and abstract screenings

Two authors (JPW and FR) independently screened the titles of all articles. Studies were included if A) the title was suggestive, or B) the title indicated that the study was conducted on humans or the subjects were not specified in the title. If human population was determined by criteria B, the study was retained if the human population was not a group currently undergoing intense medical interventions, such as chemotherapy or dialysis. Article titles that indicated that human subjects had a medical condition (e.g., diabetes, cancer) were retained during the title screening.

JPW and FR then also conducted an independent abstract screening. Articles were retained if the abstract did not specify the study population, or when the abstract noted the subjects were humans that were *not* currently undergoing intense medical interventions or there was no mention of medical interventions. Articles were also retained if the human population did not have a condition that adversely affects zinc status, such as pancreatic insufficiency or inflammatory bowel diseases [22]. Studies that were conducted only with diabetic patients (without non-diabetic control group) were excluded at this stage since studies of diabetic patients only would not contain sufficient variation in glucose levels to estimate the OR between ZD and high FPG, as there would be no subjects with normal FPG levels. To ensure that the results of the systematic review and meta-analysis would be analogous to the DALYs produced by the Institute of Health Metrics and Evaluation (IHME), studies were retained only if glucose (fasting or non-fasting) was measured; studies that only measured other biomarkers related to FPG (e.g., HbA1c) were not retained. No exclusion was made based on the zinc indicator noted in the abstract, and studies measuring zinc status (e.g., serum/plasma zinc, toenail zinc, hair zinc) and dietary zinc intake were all retained.

#### Full text reviews and data extraction

As part of the full text reviews, all relevant data were extracted into a Microsoft Excel worksheet. The full text reviews and data extraction were undertaken by two researchers (FR & WESD). A third researcher (JPW) extracted the data from a random subsample for 20% of the studies. A full double data extraction was planned if the kappa value of the inter-observer agreement was below 0.6 [23], but this was not required as the kappa value was 0.85.

As part of the data extraction, general information was recorded about the design of the studies and the population groups included. Regarding zinc status, information related to zinc biomarkers was collected, such as the unit of measurement, deficiency cutoff used, and ranges of zinc if presented as quantiles. Information about zinc intake was also collected, including the measurement method and cutoff for insufficient intake. Basic information related to glucose was also recorded, including the glucose indicator, measurement unit and cutoffs used. The researchers noted if any association between zinc and glucose was presented in the article, and if so, noted the metric that was presented (e.g., OR, risk ratio, hazard ratio) and if it was calculated using bivariate or multivariate models. When multivariate models were used, the control variables were also recorded.

During the data extraction process, the research team also noted if a study did not report the outcomes in a useable manner, but contained variables of interest (e.g., plasma zinc and FPG). For these articles, the research team contacted the respective corresponding authors by email and requested that the data be reanalyzed to produce the required ORs or for the raw data to be provided. When sending the requests, the research team asked for authors to conduct an unweighted logistic regression with selected control variables (e.g., age, sex, body-mass index, smoking status) if the study had a cross-sectional, case-control, or cohort design. For RCTs, the research team requested that a conditional logistic regression be used to generate the required ORs.

#### Publicly available data and primary data analysis

An ancillary activity of the systematic review was an online search for publicly available data that could be analyzed for the purposes of this study. The authors were able to identify and obtain datafiles from the US NHANES and the South Korea NHANES (KNHANES). Regarding the US-NHANES, three survey rounds contained both zinc and glucose data: 2011–12, 2013–14, and 2015–16. As the systematic review already identified an article including data from the 2011–12 and 2013–14 rounds [24], only the 2015–2016 US-NHANES data were utilized in addition. Regarding the KNHANES, data from 2010 contained both zinc and glucose measures [25]. The 2010 KNHANES dataset was not, however, included in the meta-analysis as the regression results were unstable because only four of the 2354 subjects with both zinc and glucose data were zinc deficient.

#### Risk of bias

Risk of bias was assessed for all studies identified through the systematic review and all primary databases obtained. To assess the risk for bias, we used a modified version of the Newcastle Ottawa Scale (NOS) for evaluating the quality of non-randomized studies in meta-analyses [26]. The modification consisted mainly in expanding the NOS to include cross-sectional studies and specific features of our analysis. Specific characteristics that were assessed included representativeness, randomness, ascertainment of exposure (i.e., indicator used to assess ZD), and ascertainment of outcomes (i.e., analytical method used to measure FPG concentration). The quality of the studies included were rated good, fair, or poor depending on the score given to each characteristic following the guidelines of the NOS. Two researchers (WZ & JPW) independently reviewed the risk of bias of each study to be included in the meta-analyses.

### Ethical considerations

As primary data collection was not required for this study, no ethical approval was sought. Prior to conducting the study, the study protocol was registered with the international registry of systematic reviews, PROSPERO (CRD42021264234).

### Laboratory analyses

Zinc concentrations were measured using atomic absorption spectrometry by Gonoodi *et al*. [27], Bo *et al*. [28], and Obeid *et al*. [29], and using inductively coupled plasma mass spectrometry by Shan *et al*. [30], Bulka *et al*. [24], Li *et al*. [31], and the 2015–16 NHANES [32]. Bulka *et al*. [24] and the 2015–16 NHANES [33] measured FPG concentrations using the “gold standard” hexokinase enzymatic method. Bo *et al*. [28] measured FPG concentrations using the glucose oxidase method, whereas all other studies use automatic biochemical analyzers or commercial kits for measuring FPG.

### Statistical analysis

#### Indicator parameters

ZD was pre-defined as serum/plasma zinc ≤70 ug/dL [34] when raw data were available or when authors re-analyzed data for this study. When data were not presented in a dichotomous format, a conversion was made to indirectly estimate the dichotomous association between ZD and high FPG with a modelled threshold of ≤70 ug/dL (see details below). High FPG glucose concentration was defined as ≥ 126 mg/dl based on the WHO diagnostic criteria for diabetes [35].

#### Primary data analysis of 2015–16 NHANES

Primary data analysis of the 2015–16 NHANES survey was done using a similar approach as used by Bulka *et al*. [24], who used data from the NHANES’ 2011–12 and 2013–14 rounds and whose results were included in the meta-analysis. In brief, we developed a logistic regression model with high FPG as the dependent variable. The independent variables in the model included ZD, age, BMI, education level, total caloric intake, number of alcoholic drinks per day in the past year, smoking status, and physical activity status. The regression model accounted for stratification and clustering, but survey weights were not used for the regression as the NHANES does not recommend weighting when key indicators were measured in different sub-samples, as was the case for zinc and glucose [36].

#### Conversion of categorical results to dichotomous results

All the studies used the dichotomous outcome high FPG. ORs and 95% confidence intervals were estimated for each study. A conversion step was performed when zinc status was not measured dichotomously. If zinc status was measured in percentiles, a weight was calculated and assigned to each OR obtained from the study. The weights were calculated as 1/(number of groups -1). For example, if zinc status was grouped in terciles (three categories), two ORs and the associated 95% confidence intervals should be obtained from the study, then a weight of 0.5 was attached to each of the ORs. To achieve normality of the distribution, we then converted each OR to the logarithmic form. Using the total sample size in the study, we calculated the number of observations in the sample in each category, and then re-sampled from the transformed distributions by randomly drawing with replacement the number of log ORs equal to the sample size for each category. A weighted log OR and associated 95% CIs were then calculated as the aggregated log OR if the zinc status were measured dichotomously. The OR was then estimated by taking the antilog of log OR.

When zinc status was measured as a continuous variable, the study result was interpreted as the change in log odds with each 1-unit increase in zinc. In contrast, we used a dichotomous ZD variable (0 = not deficient, 1 = deficient) in the analysis of NHANES 2015–16 data to calculate the change in log odds of elevated FPG when comparing those with ZD to those who are not deficient. The value was determined to be 23.79 *μg/dl*. The OR for dichotomous measure was then estimated through exponentiation, as exp(ln(1/OR*23.79)).

#### Meta analyses

A total of seven studies were included in the meta-analysis (see **Fig 1**). For the meta-analysis, we estimated the weighted pooled effect size (OR) and its 95% confidence interval (CI) using a random-effects model. The selection of the model was determined by the degree of heterogeneity, which was evaluated using the I2 statistic. The random-effects model was favored in the presence of heterogeneity. To explore heterogeneity among studies, we reran the meta-analyses after we removed each study one at a time to examine its impact on the heterogeneity and summary of effect size. Additionally, raw and standardized residuals of the fitted random-effects model were examined. A forest plot was generated to present the summary of effects size. Funnel plot asymmetry was evaluated, and a significant publication bias was considered if the P value was less than 0.05. All meta-analyses were performed using the Metafor package in R statistical software (version 4.1.2; R Foundation, Vienna, Austria). All tests were 2-tailed, and P < .05 was considered statistically significant.

#### Estimating PAR

The OR produced by the meta-analysis was used in conjunction with estimates of the prevalence of high FPG at the country- or GBD *Super Region*-level to calculate PARs. The estimated prevalence of high FPG was calculated for all countries and GBD *Super Region*s using the estimated density curves for FPG that were previously calculated by the 2019 GBD project [37]. In the 2019 GBD project, for each country or region, a lower bound of 126 mg/dl was set, and the area under the curve above the lower bound was determined and used to calculate the proportion of each population with FPG ≥ 126 mg/dl or the area above the lower bound.

#### Estimation of NCD burden attributable to ZD

Prior to estimating the disease burden attributable to ZD, we modified the methodology used by IHME to estimate the disease burden from high FPG by excluding the burden of disease for the estimated proportion of the population with FPG concentrations < 126 mg/dl. This was done so that the DALYs from high FPG would match the dichotomous nature of the meta-analysis, whereby the OR expressed the association between two dichotomous variables, ZD and high FPG, where the null value of the high FPG variables presumes no risk. This is in contrast to the publicly-available DALYs attributable to high FPG available on the Global Health Data Exchange [38] that use a continuous risk curve to estimate how the burden of disease increases as glucose concentrations rise.

DALY estimates were calculated for all disease outcomes based on IHME’s hierarchy of four aggregation levels of disease burden. Movement down the hierarchy (i.e., from level 1 to level 4) results in greater specificity in disease groupings at each subsequent level. The total disease burden at lower levels aggregates to the next higher level in the hierarchy. For our purposes, IHME’s “level 3” diseases provide a suitable level of specificity for estimating the zinc-related disease burden. Specifically, “level 3” outcomes of high FPG included 15 separate conditions: 1) alzheimer’s disease and other dementias, 2) bladder cancer, 3) blindness and vision loss, 4) breast cancer, 5) chronic kidney disease, 6) colon and rectum cancer, 7) diabetes mellitus, 8) ischemic heart disease, 9) liver cancer, 10) ovarian cancer, 11) pancreatic cancer, 12) peripheral artery disease, 13) stroke, 14) tracheal, bronchus, and lung cancer, and 15) tuberculosis.

To estimate the FPG disease burden associated with ZD, the PAR for each country was multiplied by the DALYs associated with the 15 aforementioned disease outcomes for that country. The resulting product was used as the point estimate for the DALYs attributable to ZD. To estimate confidence intervals around these point estimates, Monte Carlo simulation was performed. We conducted 10,000 random draws of data on the disease-specific burden and OR for high FBG from ZD, respectively, and estimated the disease specific burden attributable to ZD from the 10,000 samples. Values at the 2.5 and 97.5 percentile were then estimated as the 95% conference interval of the point estimate.

The high FPG disease burden associated with ZD was calculated and presented both as total DALYs and population-standardized DALYs (i.e., DALYs per 100,000 people). The latter is the quotient of the total DALYs (numerator) and the IHME’s 2019 population estimate (denominator) for countries and *Super Regions* [39].

#### Geographic aggregation and visualization

This manuscript presents disease burden results at the global and *Super Region* levels. Country-level results are presented in Table A in S1 Text. The GBD project developed *Super Regions* as a way of grouping countries based on the geographical proximity and epidemiological similarity, resulting in seven groupings: 1) Sub-Saharan Africa, 2) SAEAO, 3) CEEECA, 4) Latin America and Caribbean, 5) North Africa and Middle East, 6) South Asia, and High-Income countries [40]. A list of countries assigned to each *Super Region* are provided in Table B in S1 Text.

To visualize the results of the study, all maps categories were created by creating quintiles of each variable being mapped, and the cutoffs of each group were selected to have roughly equal numbers of countries in each quintile.

#### Patient and public involvement

Our study used secondary and publicly-available data exclusively, and did not require the collection of data from patients or any study participants. As no patients or study participants were involved in the study, these groups will not be included at the time the results are released.

## Results

### Literature search

**Fig 2:**
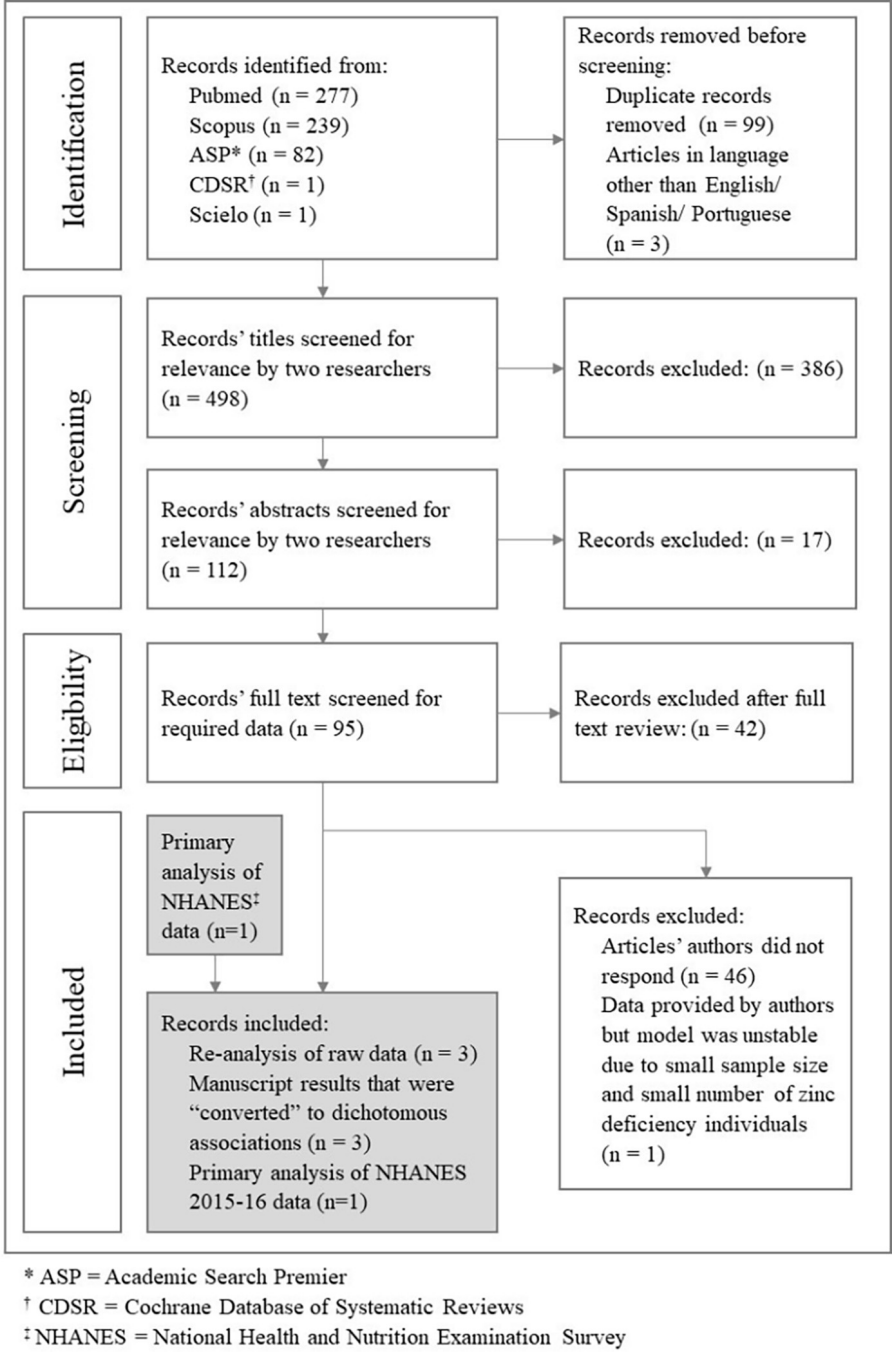
PRISMA flow chart for study selection.

We conducted a literature search to identify studies that specifically included information on both zinc status (deficient vs. sufficient) and FPG status (elevated vs. normal). After removing duplicate references found during the searches of the literature databases, there were 498 unique references included in the systematic review. As shown in **Fig 2**, the title screening eliminated 386 references, and the subsequent abstract screening eliminated an additional 17 references. As part of the full text review of 95 articles, 42 references were excluded as they did not meet the inclusion criteria. Following the full text review, three articles contained results (e.g., ORs between zinc status quantiles and high FPG) that could be converted to dichotomous associations. One of the three articles (Bulka *et al*. [24]) analyzed data from the US National Health and Nutrition Examination survey (NHANES) rounds from 2011–12 and 2013–14. Using the publicly available NHANES data from a subsequent survey round, JPW and WZ replicated the multivariable model constructed by Bulka *et al*. and conducted primary data analysis using the 2015–16 NHANES data (see details in the "Statistical Analysis–Primary data analysis of 2015–16 NHANES” below). These results constitute an additional data point for the meta-analysis.

As part of the author outreach, another three studies containing zinc and glucose status were re-analyzed and subsequently included in the analysis. Raw data was also obtained for a study by Vidović *et al*., [41] but due to the study’s small sample size (n = 60) and minimal overlap of between ZD and high FPG (n = 1), regression results were unstable and could not be included in the meta-analysis.

### Description of included studies

We conducted a meta-analysis to calculate a pooled estimate of the dichotomous association between high FPG and ZD. **Table 1** describes the basic information of the studies included in the meta-analysis. The included studies collected data on subjects in China, Iran, Italy, Lebanon, and the United States of America (USA). Only one included study used a case-control design [30], whereas the other studies were cross-sectional studies. Only the Gonoodi *et al*. [27] study was conducted on adolescents girls, whereas other studies were conducted on adults. Apart from the study by Bo *et al*. [28], which was conducted in pregnant women, all other studies contained non-pregnant women and men. All included studies used serum or plasma zinc as the zinc status indicator, and measured plasma glucose concentrations in individuals that were fasting. In total, the included studies contained 5,858 subjects.

**Table 1:** Characteristics of included studies included in Zn-FBG meta-analysis.

Study (ref), publication year	Country of data collection	Design	Participants, n	Age range or mean age in years	Sex	Covariates included in logistic regression	Conversion required (Yes/No)
Gonoodi *et al*. [27], 2018	Iran	Cross-sectional	Adolescent girls, 408	12–18	Female	Age and body-mass index categories	No
Shan *et al*. [30], 2014	China	Case-control	Adults, 1796	≥25	Both	Age, sex, body-mass index, family history of diabetes, and hypertension status	Yes
Bo *et al*. [28], 2005	Italy	Cross-sectional	Pregnant women, 194	33.0 ± 4.9	Female	Age, gestational age, body-mass index before pregnancy, and familial diabetes	Yes
Obeid *et al*. [29], 2008	Lebanon	Cross-sectional	Adults, 398	18–65	Both	Sex, age, body-mass index	No
Bulka *et al*. [24], 2019	USA	Cross-sectional	Adults, 1088	>20–80	Both	Age, sex, body-mass index, race/ethnicity, family income: poverty ratio, total caloric intake, educational attainment, smoking status, average number of drinks per day in past year, physical activity status	Yes
Li *et al*. [31], 2019	China	Cross-sectional	Adults, 1478	21–80	Both	Age, sex, area (Shimen or Huayuan), body mass index, education, smoking status, alcohol consumption status, and physical activity	No
NHANES microdata [42], 2015–2016	USA	Cross-sectional	Adults, 496	20–80	Both	Age, sex, body-mass index, race/ethnicity, family income: poverty ratio, total caloric intake, educational attainment, smoking status, average number of drinks per day in past year, physical activity status	No

The risk of bias of the identified studies is presented in **Table 2**. Three of the seven studies were classified as low risk, and the remaining four studies were classified as medium risk. No studies were excluded based on the risk of bias assessment. Only two studies were based on nationally-representative survey data, and two studies were not representative as subjects were recruited at medical facilities, and one study [31] did not specify how participants were recruited. Simple random selection of subjects was only undertaken by three studies, and the randomness of other studies was mixed. While included studies measured serum zinc using similar spectrometry methods, only two studies measured FPG concentrations with the gold standard hexokinase enzymatic method. ORs were directly estimated using logistic regression in four studies, whereas ORs from three studies were converted.

**Table 2:** Risk-of-bias assessment by study and domain. Bias rankings were based on a modified version of the Newcastle Ottawa Scale for evaluating the quality of non-randomized studies in meta-analyses [26].

Domain	Scoring criteria	Gonoodi *et al*. 2018	Shan *et al*. 2014	Bo *et al*. 2004	Bulka *et al*. 2019	Li *et al*. 2019	Obeid *et al*. 2008	NHANES 2015–16
**Representativeness**	0 = nationally representative, 1 = representative at lower administrative level, 2 = not representative	1	2	2	0	2	1	0
**Randomness of sample**	0 = Fully random, 1 = partially random, 2 = not random	0	1	1	0	2	2	0
**Biomarker of exposure (i.e., zinc status)**	0 = Serum/Plasma Zinc, 1 = Other zinc biomarker, 2 = dietary zinc	0	0	0	0	0	0	0
**Measurement method of FPG**	0 = hexokinase enzymatic method, 1 = glucose oxidase or automatic biochemical analyzers or commercial kits	1	1	1	0	1	1	0
**Measurement of association**	0 = Odds ratio directly calculated 1 = Dichotomous odds ratio converted from continuous or quantile odd/risk/hazard ratios	0	1	1	1	0	0	0
**Risk score**		2	5	5	1	5	4	0
**Risk category**	**0–3 = low risk** **4–6 = medium risk** **7–9 = high risk**	Low risk	Medium risk	Medium risk	Low risk	Medium risk	Medium risk	Low risk

### Meta analyses on the relationship between ZD and FPG

**Fig 3:**
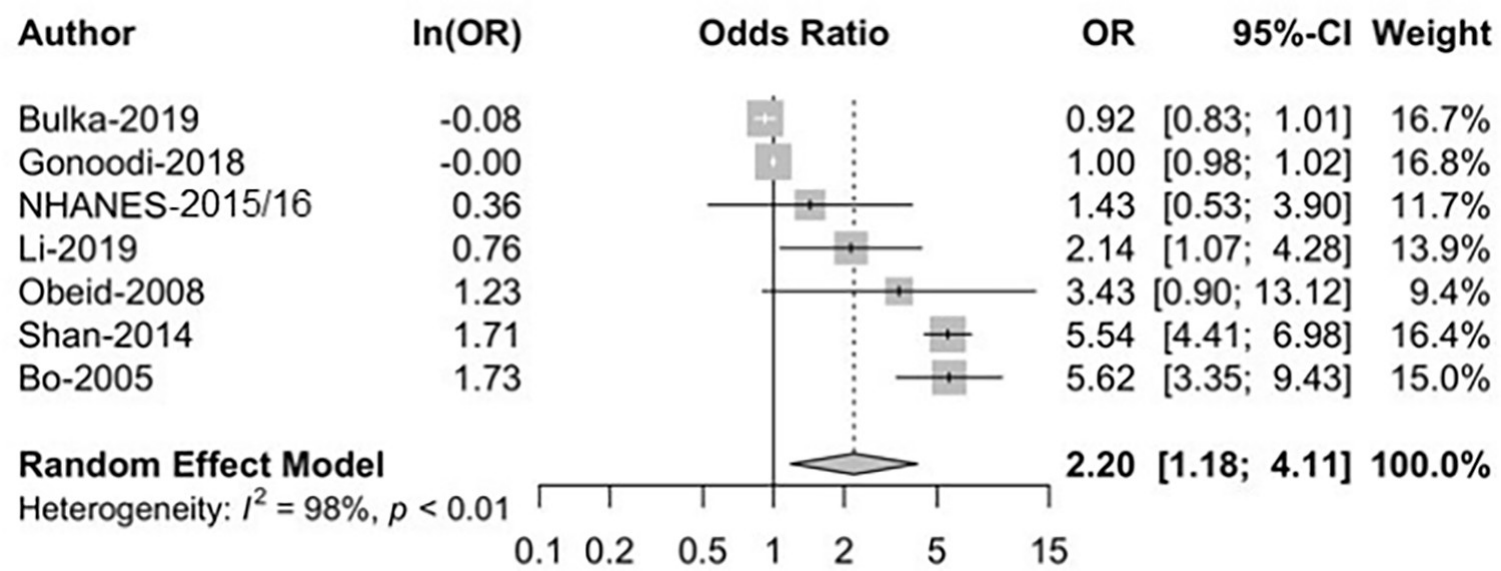
Meta-analysis of the association between zinc deficiency and high fasting plasma glucose.

Meta-analysis results are presented in **Fig 3**, and show a significant (p<0.01, random effects model) inverse relationship between ZD and high FPG. This association was observed in five of the seven studies included in the meta-analysis. The inclusion of a data point based on primary data analysis (i.e., NHANES 2015–16 data) did not substantially impact the results; the model OR based only on the six studies obtained via the systematic review was similar (OR = 2.34; 95% CI: 1.16, 4.72). Further, an examination of the standardized residuals of the fitted random-effects model showed that none of the included studies had statistically significant high residuals.

### Population proportion of high FPG attributable to ZD

Using the calculated OR from the meta-analysis and estimated prevalence of high FPG, we determined each country’s PAR of high FPG to ZD. At the global level, the PAR of ZD’s contribution to high FPG was 6.7%. For GBD *Super Regions*, the lowest PARs were found in Sub-Saharan Africa (2.4%). Higher PAR levels were found in South Asia (6.4%), North Africa and Middle East (6.5%), Southeast Asia, East Asia, and Oceania (SAEAO) (6.7%), and Central Europe, Eastern Europe, and Central Asia (CEEECA) (6.9%), and the highest PARs were found in Latin America and Caribbean (7.7%), and the GBD High-Income *Super Region* (10.5%). The PAR for all *Super Region*s and countries is provided in Table A in S1 Text.

### DALYs attributable to ZD

As shown in **Table 3**, high FPG contributes to the disease burden of 15 separate “level 3” causes by the GBD categorization. The largest share of the FPG disease burden is from “cardiovascular diseases” and “diabetes and chronic kidney diseases”, and these conditions account for more than 90% of the total FPG DALYs. Accordingly, these conditions also account for the greatest share of the disease burden attributable to ZD.

**Table 3:** DALYs from high FPG, and DALYs attributable to ZD at the global level by GBD level-3 causes, 2019*.

Level 2 Causes	Level 3 Causes	2019 DALYs†		DALYs attributable to zinc deficiency		DALYs attributable to zinc deficiency, per 100,000	
		DALYS	95% CI	DALYS	95% CI	DALYS	95% CI
**Neoplasms**		**8,575,244**	**(2,355,428, 17,557,275)**	**678,242**	**(102,065, 1,609,825)**	**8.77**	**(1.32, 20.81)**
	Bladder cancer	399,383	(81,547, 865,320)	32,388	(4,074, 83,815)	0.42	(0.05, 1.08)
	Breast cancer	1,238,619	(237,680, 2,785,554)	93,679	(13,147, 239,169)	1.21	(0.17, 3.09)
	Colon and rectum cancer	1,902,208	(453,351, 4,167,499)	151,246	(20,987, 393,517)	1.95	(0.27, 5.09)
	Liver cancer	99,249	(23,846, 217,971)	7,427	(1,007, 19,474)	0.10	(0.01, .25)
	Ovarian cancer	353,538	(68,469, 823,891)	27,483	(3,704, 71,335)	0.36	(0.05, .92)
	Pancreatic cancer	943,775	(221,091, 2,037,675)	77,165	(10,155, 199,975)	1.00	(0.13, 2.58)
	Tracheal, bronchus, and lung cancer	3,638,472	(855,701, 8,008,314)	288,852	(36,558, 739,907)	3.73	(0.47, 9.56)
**Sense organ diseases**		**672,727**	**(159,423, 1,564,433)**	**45,527**	**(5,924, 119,163)**	**0.59**	**(0.08, 1.54)**
	Blindness and vision loss	672,727	(159,423, 1,564,433)	45,527	(5,924, 119,163)	0.59	(0.08, 1.54)
**Neurological disorders**		**2,445,832**	**(413,738, 7,861,236)**	**195,891**	**(8,788, 570,701)**	**2.53**	**(0.11, 7.38)**
	Alzheimer’s disease & other dementias	2,445,832	(413,738, 7,861,236)	195,891	(8,788, 570,701)	2.53	(0.11, 7.38)
**Cardiovascular diseases**		**19,002,527**	**(13,200,142, 27,561,958)**	**1,364,478**	**(211,992, 3,262,716)**	**17.63**	**(2.74, 42.17)**
	Ischemic heart disease	11,103,141	(6,413,307, 18,357,813)	807,031	(124,350, 1,956,200)	10.43	(1.61, 25.28)
	Peripheral artery disease	429,156	(274,718, 675,697)	35,801	(5,545, 88,248)	0.46	(0.07, 1.14)
	Stroke	7,470,230	(4,608,885, 12,049,312)	521,645	(79,503, 1,266,180)	6.74	(1.03, 16.36)
**Respiratory infections and tuberculosis**		**3,800,995**	**(2,412,798, 5,199,038)**	**212,670**	**(31,639, 525,132)**	**2.75**	**(0.41, 6.79)**
	Tuberculosis	3,800,995	(2,412,798, 5,199,038)	212,670	(31,639, 525,132)	2.75	(0.41, 6.79)
**Diabetes and kidney diseases**		**82,087,721**	**(70,100,149, 95,923,086)**	**5,738,229**	**(906,207, 13,461,173)**	**74.16**	**(11.71, 173.97)**
	Diabetes mellitus	69,230,727	(58,236,244, 82,305,643)	4,847,319	(764,491, 11,388,030)	62.65	(9.88, 147.18)
	Chronic kidney disease	12,856,993	(10,875,354, 14,984,438)	890,910	(142,148, 2,092,086)	11.51	(1.84, 27.04)
**Total**	** **	**116,585,045**	**(97,195,911, 139,180,788)**	**8,235,036**	**(1,295,611, 19,182,407)**	**106.43**	**(16.74, 247.92)**

^*^ DALYs, Disability-Adjusted Life Years; FPG, Fasting Plasma Glucose; GBD, Global Burden of Disease project; ZD, Zinc Deficiency.

^**†**^ A modified version of the calculation used by IHME to estimate the disease burden from high FPG was used to estimate the 2019 DALYS. This involved excluding the burden of disease for the estimated proportion of the population with FPG concentrations < 126 mg/dl.

As shown in **Table 4**, there is considerable regional variation in the scale of the disease burden from high FPG, as well as the share of the burden attributable to ZD. South Asia, SAEAO, and High-Income countries account for more than 70% of the high FPG disease burden and zinc-attributable disease burden globally. The population-standardized disease burden (i.e., DALYs per 100,000 people) in South Asia and SAEAO is lower than all regions apart from Sub-Saharan Africa, indicating that the large population size of these regions is a key driver of the total disease burden. The population-standardized disease burden is largest in High Income countries, Latin America and the Caribbean and in CEEECA.

**Table 4:** Total DALYs*, DALYs (total and standardized) attributable to ZD by GBD *Super Region*s, 2019.

GBD *Super Region*s	2019 Population	DALYs from high FPG		DALYs attributable to ZD		DALYs attributable to ZD, per 100,000	
		DALYs	95% CI	DALYs	95% CI	DALYs	95% CI
**Central Europe, Eastern Europe, and Central Asia**	417,725,139	7,139,995	(5,629,602, 9,005,307)	496,097	(77,541, 1,182,410)	118.76	(18.56, 283.06)
**High-Income Countries**	1,083,976,063	20,792,148	(16,007,450, 26,446,113)	2,174,228	(337,965, 4,947,321)	200.58	(31.18, 456.41)
**Latin America and Caribbean**	584,378,201	11,869,848	(10,084,837, 13,881,480)	917,273	(145,722, 2,175,099)	156.97	(24.94, 372.21)
**North Africa and Middle East**	608,713,645	8,650,929	(7,491,033, 10,160,736)	561,053	(88,773, 1,347,994)	92.17	(14.58, 221.45)
**South Asia**	1,805,200,296	26,821,314	(22,672,025, 31,485,652)	1,711,731	(260,223, 4,087,413)	94.82	(14.42, 226.42)
**Southeast Asia, East Asia, and Oceania**	2,159,261,972	32,630,669	(27,034,554, 39,566,269)	2,170,135	(339,165, 5,169,313)	100.50	(31.46, 479.44)
**Sub-Saharan Africa**	1,078,209,307	8,680,141	(7,083,279, 10,374,583)	204,519	(31,782, 520,484)	18.97	(2.95, 48.27)
**Grand Total**	**7,737,464,623**	**116,585,045**	**(97,195,911, 139,180,788)**	**8,235,036**	**(1,295,611, 19,182,407)**	**106.43**	**(16.74, 247.92)**

* DALYs, Disability-Adjusted Life Years; FPG, Fasting Plasma Glucose; GBD, Global Burden of Disease project; ZD, Zinc Deficiency.

**Fig 4:**
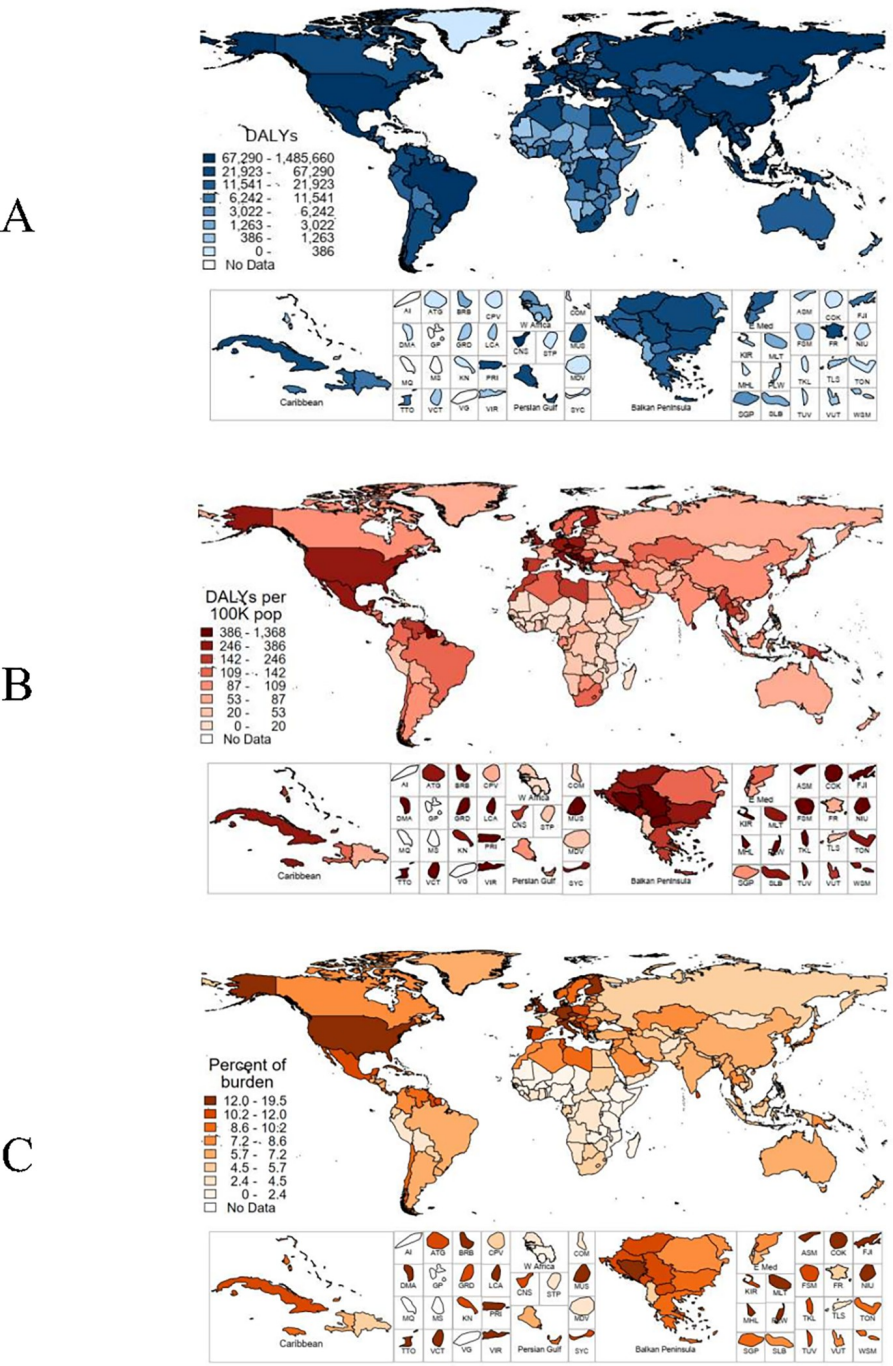
Country level non-communicable disease burden attributable to zinc deficiency: (A) total DALYs, (B) DALYs per 100,000 people, (C) Percent of high FPG disease burden attributable to zinc deficiency. (Map base layer: https://hub.arcgis.com/datasets/UIA::uia-world-countries-boundaries/about).

**Fig 4** illustrates the geographic distribution of the disease burden from high FPG that is attributable to ZD at the country level. The three countries with the largest total number of DALYs attributable to ZD (Panel A) are India (~1.5 million), China (~1.3 million), and the United States of America (USA; ~1.0 million). Countries with high population-standardized DALYS (Panel B; DALYS/100000 people) were largely found in island nations or states: Niue (1368), Palau (1231), Fiji (1072), Mauritius (1021), Cook Islands (954), American Samoa (843), Trinidad & Tobago (811), Puerto Rico (724), Marshall Islands (674), the US Virgin Islands (634), and others. Among the three countries with the highest total DALYs, the USA had the highest number of DALYs per 100,000 (309), followed by India (107) and China (89). The proportion of the high FPG disease burden attributable to ZD (Panel C) was highest among the island nations of Niue (19.5%), American Samoa (18.2%), Palau (17.4%), Cook Islands (16.4%), Puerto Rico (16.3%), and others. ZD also accounted for a relatively high proportion of the disease burden in the USA and many Western European countries. ZD accounted for the lowest proportion of the disease burden in most countries in Sub-Saharan Africa, with the lowest PAR found in Niger (1.3%).

## Discussion

### Disease burden attributable to ZD

To the authors’ knowledge, this is the first study to estimate the NCD burden attributable to ZD. Prior to our analysis, ZD’s contribution to the global disease burden, as estimated by the GBD project in 2019, has been estimated as a portion of the disease burden from diarrhea in children 1–4 years of age [43]. The GBD project previously included ZD as a risk factor of lower respiratory infection (LRI) [44], but ZD was not considered a risk factor for LRI in 2019 as multiple studies found that reductions in ZD only generated small decrease in LRI mortality [45]. Other studies also previously developed models to estimate the zinc-attributable disease burden from diarrhea, pneumonia, and stunting in children <5 years of age [19], demonstrating the evolution in knowledge of the association as more data become available.

Our results find that ZD accounts for a modest proportion of less than 10% of the NCD burden from high FPG, globally. Nonetheless, due to the large total disease burden from NCDs caused by high FPG, the “FPG DALYs” attributable to ZD are considerably higher than the diarrhea-related DALYs attributable to ZD estimated by the GBD project [43]. To illustrate, the GBD project’s 2019 disease burden estimates show that globally, ZD accounts for 0.32% of the DALYs from diarrheal diseases (diarrheal disease burden attributable to ZD = 258,813 DALYs; total diarrheal disease burden = 80,917,779) [38]. Our estimates of the FPG DALYs attributable to ZD are approximately 32 times greater. While a number of approaches can be used to value a DALY, one approach uses a standardized value of USD 1000 to present the DALY burden in terms of an economic cost that can be considered in cost-benefit analyses considering various interventions [19]. Using this standard value, the economic cost of the ZD-associated NCD burden would be currently estimated at USD 8.2 billion.

More than 90% of the FPG DALYs—and subsequently the FPG DALYs attributable to ZD—stem from four disease outcomes: diabetes mellitus, ischemic heart disease, stroke, and chronic kidney disease. The *global* disease burden from each of these disease outcomes has increased over the past 30 years, however, these disease outcomes have not increased in all regions. To illustrate, in industrialized countries, the disease burden from type 2 diabetes has steadily increased since 1990 and is predicted to continue increasing into the future [46]. In contrast, the disease burden from ischemic heart disease has declined in *High-Income* countries since 1990, and global increases were driven by an increasing disease burden in other regions, with the biggest increases found in South Asia and in SAEAO [38], which are the two most populous super-regions and have experienced a significant rise in the mortality rate from ischemic heart disease [47]. The disease burdens due to stroke and chronic kidney disease have also increased since 1990, with the majority of the increase in the burden from stroke observed in SAEAO [38], and similar increases in the total burden from chronic kidney disease in both South Asia and SAEAO. The only decreases in the four outcomes between 1990 and 2019 occurred in *High-Income* countries, where the disease burdens from ischemic heart disease and stroke decreased, and in CEEECA, where the stroke disease burden decreased.

While the disease burden is highest among populous countries (e.g., China, India, USA), the population-standardized DALYs are highest among island nations, particularly island nations in the South Pacific and Caribbean. High levels of NCDs in these regions have been previously identified. A pooled analysis of 751 population-based studies observed that Micronesia and Polynesia experienced the largest increase in the prevalence of diabetes between 1980 and 2014, with the highest prevalence in 2014 found in American Samoa [48]. Increases in the prevalence of diabetes have also been observed in Caribbean nations [49]. While these nations, which have small populations, do not contribute substantially to the total disease burden, the intra-country economic effects of the high prevalence of NCDs are well documented. The costs of treatment, hospitalization, permanent disability, premature death, and other factors all add to the economic toll caused by a high prevalence of NCDs [50].

### Zinc and the “double burden” of malnutrition

ZD alongside the sizable disease burden from NCDs denotes a “double burden” of malnutrition, where under- and over-nutrition occurs in the same communities and/or individuals [51]. However, while a “double burden” simply implies co-occurrence of measures of under- and over-nutrition [52], ZD appears to directly contribute to the disease outcomes (e.g., diabetes, heart disease) typically associated with over-nutrition outcomes, such as overweight and obesity. Thus, a “double burden” of malnutrition when ZD is the indicator of under-nutrition characterizes a situation whereby a portion of the over-nutrition disease burden could be reduced by programs that increase the intake of zinc and decrease the prevalence of ZD.

The features of this “double burden” vary by geographic region due to industrialization and dietary factors. Using data from national food balance sheets, Wessells and Brown [53] estimated that 17% of the global population was at risk of insufficient zinc intake, with prevalences <15% in most countries in North America and Western Europe, and prevalences 15%-25% or >25% in most countries on the African continent and in South and Southeast Asia. These estimates suggest that zinc interventions could be implemented in nearly all regions, including low- and middle-income countries. Though the disease burden from high FPG is lowest in Sub-Saharan Africa, many countries in this region are undergoing a “nutrition transition” where there is an increasing prevalence of conditions associated with over-nutrition and/or poor diets [2]. A similar transition is taking place in South Asia and SAEAO, and large populations in tandem with sizable FPG disease burden per capita suggest that zinc interventions would be appropriate. In addition, zinc interventions are also appropriate in *High-Income* countries, as the even modest reductions in ZD prevalence could translate to substantial reductions in the disease burden from high FPG.

### Zinc-related public health programs

The three most-common public health measures used to increase zinc intake are supplementation, industrial fortification, and biofortification. Zinc supplementation has been found to both reduce the incidence of diarrhea in children <5 years of age [54] and improve the indicators related to diabetes and cardiovascular diseases (e.g., FPG, HbA1c, insulin resistance, and LDL cholesterol) [7]. Zinc supplementation of pregnant women with low zinc status has also been shown to significantly increase the birth weight and head circumference of the offspring [55]. At present, zinc supplements are frequently administered to manage acute diarrhea in children, and the WHO recommends that children receive zinc supplements (<6 months, 10mg; 6–59 months, 20mg) for 10–14 days after a diarrheal episode [56]. Mass zinc supplementation of adults is currently undertaken as part of national prenatal programs that deliver multiple micronutrient supplements to pregnant women. Some countries have replaced iron-folic acid supplements targeted to pregnant women with multiple micronutrient supplements, as multiple micronutrient supplements have been found to lower the risk of certain birth outcomes, such as low birth weight, small-for-gestational age, and pre-term birth [57].

The addition of zinc to industrially milled cereal grains (e.g., wheat, maize, rice) is mandated in 34 countries located in Sub-Saharan Africa (n = 16), SAEAO (n = 6), Latin America and Caribbean (n = 6), CEEECA (n = 3), and North Africa and Middle East (n = 3) [58]. A recent systematic review and meta-analysis of efficacy and effectiveness studies found that that fortification with zinc and other micronutrients was associated with improved plasma zinc concentrations and increased weight gain in children [59]. Though relatively few countries fortify with zinc, there are an additional 57 countries that mandate the fortification of wheat, maize, or rice, but do not mandate the inclusion of zinc. Changes to the fortification standards in these countries could be a possible approach for governments to reduce ZD and the disease burden from NCDs at relatively low additional cost. While these findings suggest that mass fortification may be a feasible approach to delivering zinc, studies examining the coverage of fortified foods have identified issues related to compliance, resulting in a lower-than-anticipated coverage [60,61].

Zinc-biofortified crops offer another option to increase the intake of zinc in populations experiencing a high prevalence of deficiency. There are several studies with women and children that demonstrate higher total absorbed zinc from biofortified crops (e.g., pearl millet, rice, wheat, maize). However, to date there are only two published intervention trials examining the efficacy of a zinc biofortified crop. Sazawal *et al*. [62] and Jongstra *et al*. [63] conducted a double masked randomized, controlled trials in India and Bangladesh, respectively. Sazawal *et al*. [62] examined the impact of six-month consumption of biofortified wheat flour on children and mothers, and found no significant treatment effects on mean plasma zinc at endline or changes from baseline to endline. Both mothers and children consuming zinc-biofortified wheat did, however, report significantly less morbidity; mothers reported significantly fewer days with fever, and children reported significantly fewer days with pneumonia and vomiting. Jongstra *et al*. [63] examined the impact of nine-months of zinc-biofortified rice consumption in children. The researchers also found no significant changes in plasma zinc concentration, but in contrast to Sazawal *et al*. [62], found that children consuming biofortified rice had higher levels of morbidity from respiratory infections while also experiencing higher linear growth during the intervention period.

Due to the differing programmatic landscapes in industrialized and developing countries, policy makers in each country must determine the type of program(s) that best suit their population’s consumption patterns. As the total and population-standardized FPG disease burden attributable to ZD is highest among high-income countries, it is warranted to examine the feasibility of zinc interventions for these countries, particularly among elderly populations where the prevalence of ZD has been shown to be higher than the general population [64].

As the proportion of the NCD burden attributable to ZD is relatively small, programs that increase zinc intake should be implemented as part of national public health policies and programs that aim to improve a populations diet and reduce other risk factors, such as obesity, sedentarism, and hypertension.

### Strengths and limitations

The approach taken by the research team has notable strengths. As this approach utilizes a modified version of the FPG disease burden estimated annually by the GBD project, the results are truly global and would enable policymakers to identify countries where zinc interventions could be implemented to reduce the FPG disease burden attributable to ZD. Furthermore, as more data become available, the meta-analysis could be readily updated and new estimates produced.

The meta-analysis component of our study was limited by the identification of a small number of data points. This was due to the fact that few studies that measure both zinc and FPG concentrations calculated the dichotomous association required for our meta-analysis or calculated a metric that could be converted to a dichotomous OR. Furthermore, no data included in the meta-analysis were present from countries in Latin America, Sub-Saharan Africa, and Central Asia, or low-income countries in any region. While the results of our study suggest a biological association between zinc and glucose status, data from a wider array of countries would be more ideal since the meta-analysis’ OR is multiplied by national and regional estimates of the proportion of high FPG to calculate the PAR. The meta-analysis included several studies with medium risk, which would affect the accuracy of the estimation of the disease burden attributable to ZD. An obvious concern that most of the studies included in the meta-analysis were from non-representative samples with non-randomly selected participants. Future studies on this topic should be representative and should randomly select participants to provide a more accurate estimation of the strength of the relationship between ZD and high FPG. The meta-analysis portion of our study is also limited by the variation in the covariates used by each study’s regression model. Our study is also limited by the poor accuracy and precision of serum/plasma zinc. While there are distinct challenges to measuring ZD, population-level ZD is often defined as serum/plasma zinc concentrations that are below set thresholds, and serum/plasma zinc is often measured as part of population-based micronutrient and nutrition surveys [34]. Until improved biomarkers of zinc status are developed and verified, the associations between zinc status defined by serum/ plasma zinc levels and risk factors of key NCDs will have to be interpreted with caution.

## Conclusion

Via a systematic review and meta-analysis, our study found a significant association between ZD and high FPG. Calculations using the OR from the meta-regression and estimates of the disease burden from high FPG show that ZD accounts for a modest proportion of the DALYs from high FPG. While ZD accounts for a small share of the high FPG disease burden, the total number of DALYs far surpasses other estimates of the disease burden attributable to ZD, which focus on diarrheal diseases in childhood. Our results show that the disease burden from ZD is largest in *High-Income* countries, but smaller countries, particularly island nations, can have the highest per-capita disease burden.

## Supporting information

S1 Checklist The PRISMA checklist for the study can found in the supplementary file. (DOCX)

S1 Text Fig A. ZD-LDL Systematic Review PRISMA Flow Chart. Fig B. Low density lipoprotein- fasting plasma glucose Meta-Analysis results. Table A. Country-level results for high fasting plasma glucose (FPG) Disability-Adjusted Life Years (DALYs), DALYs attributable to zinc deficiency (ZD), DALYs attributable to ZD per 100,000, and Population Attributable Risk (PAR). Table B. Countries in each Global Burden of Disease Super Region.
(DOCX)
