# Supplementary material for: The global burden of high fasting plasma glucose associated with zinc deficiency: Results of a systematic review and meta-analysis
Source: PLOS Glob Public Health. 2023 Mar 13;3(3):e0001353. doi: 10.1371/journal.pgph.0001353 (PMC10022216; doi:10.1371/journal.pgph.0001353)
Supplement: S1 Checklist — (DOCX) [file pgph.0001353.s001.docx]

| **Section and Topic** | **Item #** | **Checklist item** | **Location where item is reported** |
| --- | --- | --- | --- |
| **TITLE** | | |  |
| Title | 1 | Identify the report as a systematic review. | Title; page 1 |
| **ABSTRACT** | | |  |
| Abstract | 2 | See the PRISMA 2020 for Abstracts checklist. | Page 2 |
| **INTRODUCTION** | | |  |
| Rationale | 3 | Describe the rationale for the review in the context of existing knowledge. | Rationale and objectives included in introduction; page 4 |
| Objectives | 4 | Provide an explicit statement of the objective(s) or question(s) the review addresses. | Rationale and objectives included in introduction; page 4 |
| **METHODS** | | |  |
| Eligibility criteria | 5 | Specify the inclusion and exclusion criteria for the review and how studies were grouped for the syntheses. | In sub-section entitled “Inclusion and exclusion criteria of title and abstract screenings”, page 5 |
| Information sources | 6 | Specify all databases, registers, websites, organisations, reference lists and other sources searched or consulted to identify studies. Specify the date when each source was last searched or consulted. | In sub-sections entitled “Search Strategy” and “Publicly available data and primary data analysis”; page 5 |
| Search strategy | 7 | Present the full search strategies for all databases, registers and websites, including any filters and limits used. | In sub-section entitled “Search Strategy”; page 5 |
| Selection process | 8 | Specify the methods used to decide whether a study met the inclusion criteria of the review, including how many reviewers screened each record and each report retrieved, whether they worked independently, and if applicable, details of automation tools used in the process. | In sub-section entitled “Inclusion and exclusion criteria of title and abstract screenings”, pages 5 |
| Data collection process | 9 | Specify the methods used to collect data from reports, including how many reviewers collected data from each report, whether they worked independently, any processes for obtaining or confirming data from study investigators, and if applicable, details of automation tools used in the process. | In sub-section entitled “Inclusion and exclusion criteria of title and abstract screenings” and “Full text reviews and data extraction”, page 5 & 6 |
| Data items | 10a | List and define all outcomes for which data were sought. Specify whether all results that were compatible with each outcome domain in each study were sought (e.g. for all measures, time points, analyses), and if not, the methods used to decide which results to collect. | In sub-section entitled “Experimental Design”, page 5 |
|  | 10b | List and define all other variables for which data were sought (e.g. participant and intervention characteristics, funding sources). Describe any assumptions made about any missing or unclear information. | In sub-section entitled “Inclusion and exclusion criteria of title and abstract screenings”, page 5 |
| Study risk of bias assessment | 11 | Specify the methods used to assess risk of bias in the included studies, including details of the tool(s) used, how many reviewers assessed each study and whether they worked independently, and if applicable, details of automation tools used in the process. | In sub-section entitled “Risk of bias”, page 6 |
| Effect measures | 12 | Specify for each outcome the effect measure(s) (e.g. risk ratio, mean difference) used in the synthesis or presentation of results. | In sub-sections entitled “Introduction+” and “Conversion of categorical results to dichotomous results”, page 4 & 7 |
| Synthesis methods | 13a | Describe the processes used to decide which studies were eligible for each synthesis (e.g. tabulating the study intervention characteristics and comparing against the planned groups for each synthesis (item #5)). | In sub-section entitled “Inclusion and exclusion criteria of title and abstract screenings”, page 5 |
|  | 13b | Describe any methods required to prepare the data for presentation or synthesis, such as handling of missing summary statistics, or data conversions. | In sub-sections entitled “Conversion of categorical results to dichotomous results”, pages 8 |
|  | 13c | Describe any methods used to tabulate or visually display results of individual studies and syntheses. | In sub-sections entitled “Meta-analysis” and “Geographic aggregation and visualization”, pages 8 & 9  “, page 8 and |
|  | 13d | Describe any methods used to synthesize results and provide a rationale for the choice(s). If meta-analysis was performed, describe the model(s), method(s) to identify the presence and extent of statistical heterogeneity, and software package(s) used. | In sub-sections entitled “Meta-analysis”; page 8 |
|  | 13e | Describe any methods used to explore possible causes of heterogeneity among study results (e.g. subgroup analysis, meta-regression). | In sub-sections entitled “Meta-analysis”; page 8 |
|  | 13f | Describe any sensitivity analyses conducted to assess robustness of the synthesized results. | Not applicable |
| Reporting bias assessment | 14 | Describe any methods used to assess risk of bias due to missing results in a synthesis (arising from reporting biases). | In sub-section entitled “Risk of bias”, page 6 |
| Certainty assessment | 15 | Describe any methods used to assess certainty (or confidence) in the body of evidence for an outcome. | Not applicable |
| **RESULTS** | | |  |
| Study selection | 16a | Describe the results of the search and selection process, from the number of records identified in the search to the number of studies included in the review, ideally using a flow diagram. | In sub-section entitled “Literature search”, page 9 |
|  | 16b | Cite studies that might appear to meet the inclusion criteria, but which were excluded, and explain why they were excluded. | In sub-section entitled “Description of included studies”, page 10 |
| Study characteristics | 17 | Cite each included study and present its characteristics. | In table 1, page 11 |
| Risk of bias in studies | 18 | Present assessments of risk of bias for each included study. | In table 2, page 12 |
| Results of individual studies | 19 | For all outcomes, present, for each study: (a) summary statistics for each group (where appropriate) and (b) an effect estimate and its precision (e.g. confidence/credible interval), ideally using structured tables or plots. | In Figure 2 |
| Results of syntheses | 20a | For each synthesis, briefly summarise the characteristics and risk of bias among contributing studies. | In table 2, page 12 |
|  | 20b | Present results of all statistical syntheses conducted. If meta-analysis was done, present for each the summary estimate and its precision (e.g. confidence/credible interval) and measures of statistical heterogeneity. If comparing groups, describe the direction of the effect. | In sub-section entitled “Meta analyses on the relationship between ZD and FPG”, page 13 |
|  | 20c | Present results of all investigations of possible causes of heterogeneity among study results. | Not applicable |
|  | 20d | Present results of all sensitivity analyses conducted to assess the robustness of the synthesized results. | Not applicable |
| Reporting biases | 21 | Present assessments of risk of bias due to missing results (arising from reporting biases) for each synthesis assessed. | In table 2, page 12 |
| Certainty of evidence | 22 | Present assessments of certainty (or confidence) in the body of evidence for each outcome assessed. | Not applicable |
| **DISCUSSION** | | |  |
| Discussion | 23a | Provide a general interpretation of the results in the context of other evidence. | Discussion section, pages 17-19 |
|  | 23b | Discuss any limitations of the evidence included in the review. | Discussion section, pages 17-19 |
|  | 23c | Discuss any limitations of the review processes used. | In sub-section entitled “Strengths and limitations”, page 13 |
|  | 23d | Discuss implications of the results for practice, policy, and future research. | In sub-section entitled “Zinc-related public health programs”, page 17-18 |
| **OTHER INFORMATION** | | |  |
| Registration and protocol | 24a | Provide registration information for the review, including register name and registration number, or state that the review was not registered. | In sub-section entitled “Ethical considerations”, page 7 |
|  | 24b | Indicate where the review protocol can be accessed, or state that a protocol was not prepared. | In sub-section entitled “Ethical considerations”, page 7 |
|  | 24c | Describe and explain any amendments to information provided at registration or in the protocol. | Not applicable |
| Support | 25 | Describe sources of financial or non-financial support for the review, and the role of the funders or sponsors in the review. | In “Funding” statement, page 24 |
| Competing interests | 26 | Declare any competing interests of review authors. | In “Competing Interests” statement, page 24 |
| Availability of data, code and other materials | 27 | Report which of the following are publicly available and where they can be found: template data collection forms; data extracted from included studies; data used for all analyses; analytic code; any other materials used in the review. | In “Data and materials availability” statement, page 24 |

*From:*  Page MJ, McKenzie JE, Bossuyt PM, Boutron I, Hoffmann TC, Mulrow CD, et al. The PRISMA 2020 statement: an updated guideline for reporting systematic reviews. BMJ 2021;372:n71. doi: 10.1136/bmj.n71

For more information, visit: <http://www.prisma-statement.org/>
