# Supplementary material for: The global burden of high fasting plasma glucose associated with zinc deficiency: Results of a systematic review and meta-analysis
Source: PLOS Glob Public Health. 2023 Mar 13;3(3):e0001353. doi: 10.1371/journal.pgph.0001353 (PMC10022216; doi:10.1371/journal.pgph.0001353)
Supplement: S1 Text — Fig A. ZD-LDL Systematic Review PRISMA Flow Chart. Fig B. Low density lipoprotein- fasting plasma glucose Meta-Analysis results. Table A. Country-level results for high fasting plasma glucose (FPG) Disability-Adjusted Life Years (DALYs), DALYs attributable to zinc deficiency (ZD), DALYs attributable to ZD per 100,000, and Population Attributable Risk (PAR). Table B. Countries in each Global Burden of Disease Super Region. (DOCX) [file pgph.0001353.s002.docx]

Supplementary Materials for

**The global burden of high fasting plasma glucose associated with zinc deficiency: results of a systematic review and meta-analysis**

James P Wirth,* Wu Zeng, Nicolai Petry, Fabian Rohner, Scott Glenn, William E.S. Donkor, Rita Wegmüller, Erick Boy, Keith Lividini

*Corresponding author. Email: james@groundworkhealth.org

Fig. A. ZD-LDL Systematic Review PRISMA Flow Chart

**
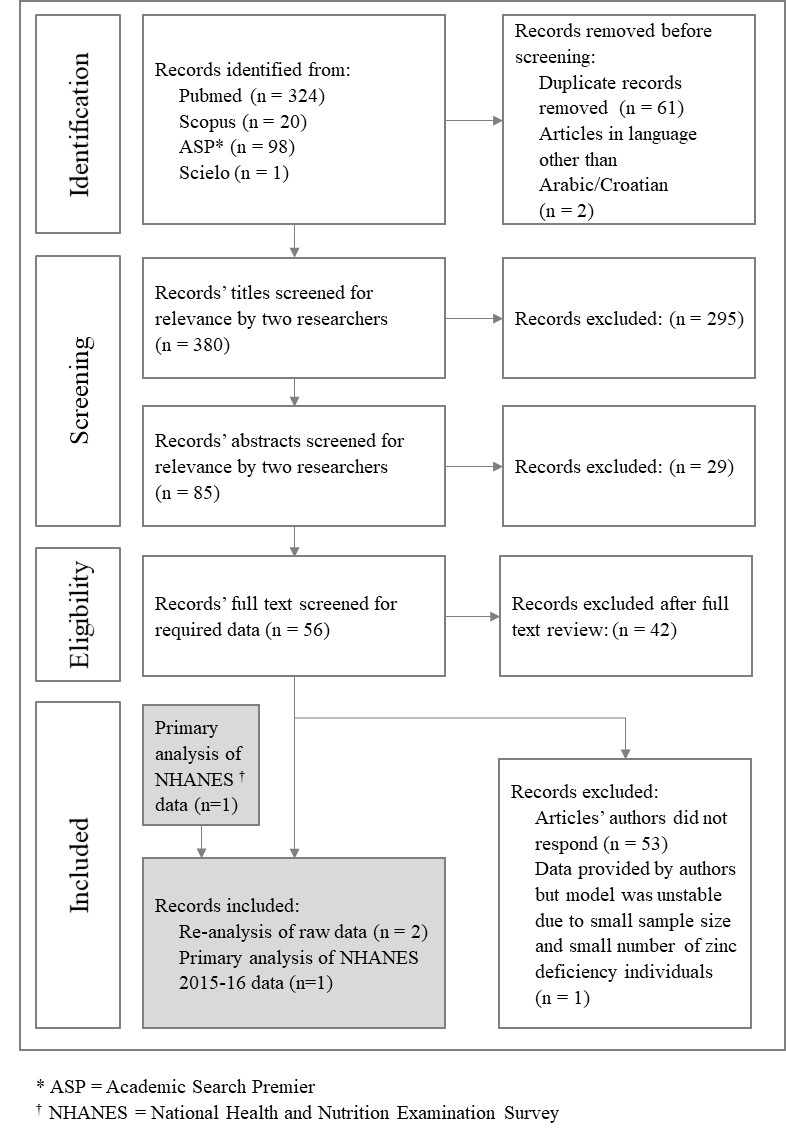
**

Fig. B. Low density lipoprotein- fasting plasma glucose Meta-Analysis results.


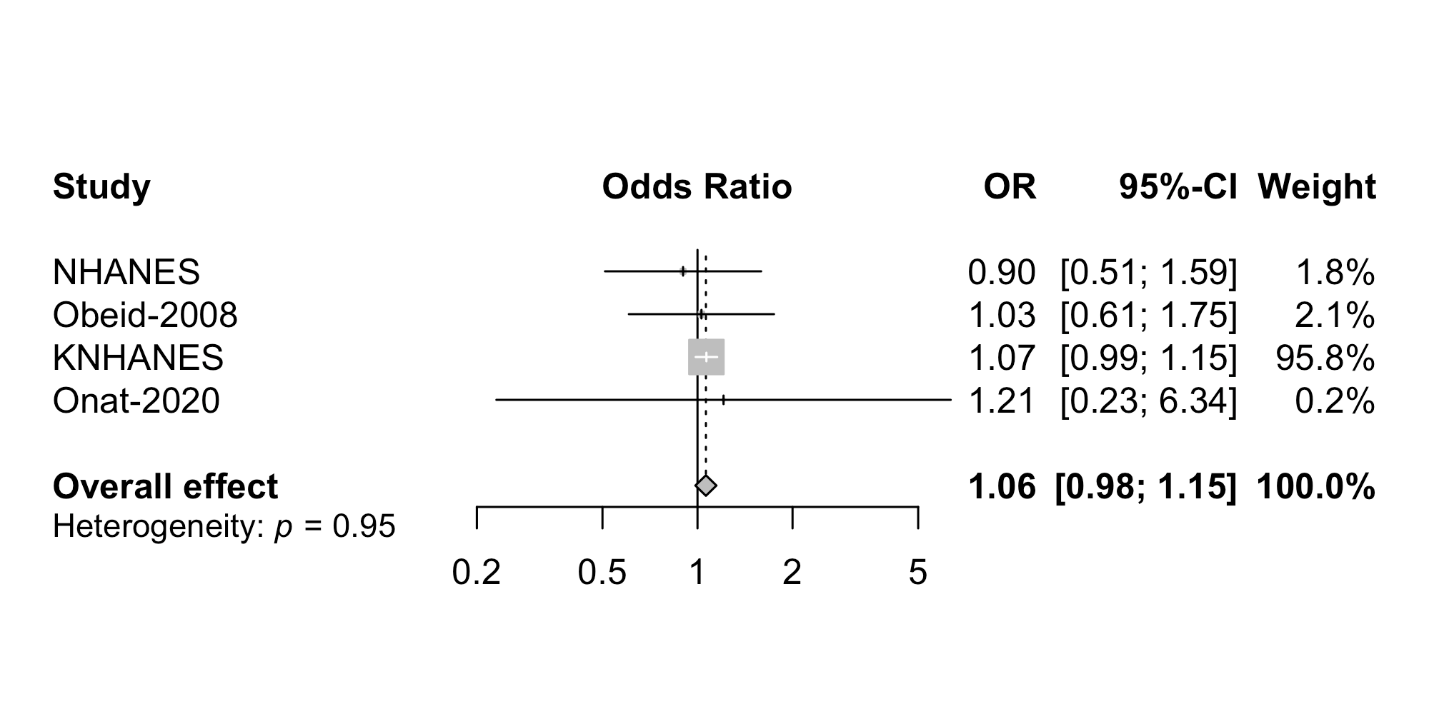


Table A. Country-level results for high fasting plasma glucose (FPG) Disability-Adjusted Life Years (DALYs), DALYs attributable to zinc deficiency (ZD), DALYs attributable to ZD per 100,000, and Population Attributable Risk (PAR).

| **Country name** | **High FPG DALYs** | **DALYs attributable to ZD** | **DALYs attributable to ZD per 100,000** | **PAR** |
| --- | --- | --- | --- | --- |
| Afghanistan | 408,296 | 18,178 | 47 | 4.45% |
| Albania | 25,448 | 1,430 | 53 | 5.62% |
| Algeria | 618,874 | 48,377 | 116 | 7.82% |
| Andorra | 1,016 | 83 | 100 | 8.19% |
| Angola | 256,840 | 7,438 | 25 | 2.90% |
| Antigua and Barbuda | 2,832 | 308 | 348 | 10.88% |
| Argentina | 713,717 | 47,998 | 106 | 6.73% |
| Armenia | 75,979 | 6,262 | 207 | 8.24% |
| Australia | 291,393 | 19,705 | 80 | 6.76% |
| Austria | 153,114 | 12,719 | 143 | 8.31% |
| Azerbaijan | 194,581 | 12,669 | 123 | 6.51% |
| Bahamas | 9,545 | 910 | 241 | 9.54% |
| Bahrain | 38,670 | 5,365 | 372 | 13.87% |
| Bangladesh | 1,726,925 | 84,288 | 53 | 4.88% |
| Barbados | 12,548 | 1,573 | 528 | 12.53% |
| Belarus | 78,350 | 3,946 | 42 | 5.04% |
| Belgium | 177,020 | 15,225 | 133 | 8.60% |
| Belize | 7,641 | 441 | 108 | 5.77% |
| Benin | 90,436 | 2,108 | 17 | 2.33% |
| Bhutan | 9,062 | 462 | 61 | 5.10% |
| Bolivia (Plurinational State of) | 189,206 | 8,425 | 70 | 4.45% |
| Bosnia and Herzegovina | 133,156 | 17,347 | 526 | 13.03% |
| Botswana | 47,060 | 2,144 | 92 | 4.56% |
| Brazil | 3,635,337 | 240,729 | 111 | 6.62% |
| Brunei Darussalam | 11,146 | 1,284 | 294 | 11.52% |
| Bulgaria | 233,662 | 23,872 | 344 | 10.22% |
| Burkina Faso | 170,926 | 3,850 | 17 | 2.25% |
| Burundi | 87,170 | 1,661 | 14 | 1.91% |
| Côte d'Ivoire | 208,046 | 5,168 | 20 | 2.48% |
| Cabo Verde | 7,439 | 366 | 65 | 4.92% |
| Cambodia | 261,238 | 12,840 | 77 | 4.91% |
| Cameroon | 261,910 | 6,020 | 21 | 2.30% |
| Canada | 511,697 | 37,662 | 103 | 7.36% |
| Central African Republic | 84,415 | 2,958 | 56 | 3.50% |
| Chad | 103,826 | 2,008 | 12 | 1.93% |
| Chile | 286,252 | 24,724 | 136 | 8.64% |
| China | 17,669,005 | 1,272,352 | 89 | 7.20% |
| Colombia | 712,371 | 59,499 | 125 | 8.35% |
| Comoros | 7,514 | 200 | 28 | 2.67% |
| Congo | 69,871 | 2,831 | 54 | 4.05% |
| Costa Rica | 75,131 | 6,692 | 142 | 8.91% |
| Croatia | 119,916 | 13,660 | 322 | 11.39% |
| Cuba | 278,523 | 32,430 | 286 | 11.64% |
| Cyprus | 25,678 | 2,561 | 195 | 9.97% |
| Czechia | 362,719 | 57,461 | 540 | 15.84% |
| Democratic People's Republic of Korea | 368,914 | 22,427 | 85 | 6.08% |
| Democratic Republic of the Congo | 789,180 | 24,279 | 28 | 3.08% |
| Denmark | 84,065 | 6,441 | 111 | 7.66% |
| Djibouti | 11,230 | 295 | 25 | 2.63% |
| Dominica | 2,826 | 381 | 555 | 13.49% |
| Dominican Republic | 167,370 | 7,929 | 73 | 4.74% |
| Ecuador | 275,968 | 15,649 | 89 | 5.67% |
| Egypt | 1,558,439 | 78,825 | 80 | 5.06% |
| El Salvador | 149,616 | 11,871 | 190 | 7.93% |
| Equatorial Guinea | 11,574 | 347 | 24 | 3.00% |
| Eritrea | 62,246 | 1,419 | 21 | 2.28% |
| Estonia | 17,895 | 1,318 | 100 | 7.36% |
| Eswatini | 27,165 | 1,103 | 97 | 4.06% |
| Ethiopia | 578,697 | 9,071 | 8 | 1.57% |
| Fiji | 65,016 | 9,770 | 1,072 | 15.03% |
| Finland | 111,872 | 13,633 | 246 | 12.19% |
| France | 621,550 | 35,256 | 53 | 5.67% |
| Gabon | 31,102 | 1,538 | 88 | 4.94% |
| Gambia | 17,881 | 404 | 18 | 2.26% |
| Georgia | 118,358 | 12,476 | 340 | 10.54% |
| Germany | 2,063,282 | 280,313 | 330 | 13.59% |
| Ghana | 356,643 | 11,541 | 37 | 3.24% |
| Greece | 203,275 | 19,797 | 192 | 9.74% |
| Grenada | 3,850 | 439 | 425 | 11.40% |
| Guatemala | 359,206 | 24,913 | 140 | 6.94% |
| Guinea | 104,264 | 2,266 | 18 | 2.17% |
| Guinea-Bissau | 18,653 | 444 | 23 | 2.38% |
| Guyana | 29,897 | 3,149 | 409 | 10.53% |
| Haiti | 255,580 | 17,277 | 139 | 6.76% |
| Honduras | 145,199 | 9,572 | 98 | 6.59% |
| Hungary | 291,878 | 32,997 | 341 | 11.31% |
| Iceland | 3,578 | 268 | 78 | 7.49% |
| India | 21,649,930 | 1,485,660 | 107 | 6.86% |
| Indonesia | 5,580,111 | 254,668 | 98 | 4.56% |
| Iran (Islamic Republic of) | 1,202,713 | 85,547 | 101 | 7.11% |
| Iraq | 673,201 | 44,217 | 105 | 6.57% |
| Ireland | 56,769 | 4,182 | 85 | 7.37% |
| Israel | 125,959 | 8,351 | 90 | 6.63% |
| Italy | 1,400,721 | 169,705 | 281 | 12.12% |
| Jamaica | 92,517 | 9,232 | 328 | 9.98% |
| Japan | 1,801,516 | 144,808 | 113 | 8.04% |
| Jordan | 135,617 | 8,218 | 71 | 6.06% |
| Kazakhstan | 303,495 | 21,923 | 119 | 7.22% |
| Kenya | 334,189 | 6,762 | 13 | 2.02% |
| Kiribati | 6,854 | 716 | 604 | 10.45% |
| Kuwait | 50,353 | 4,941 | 112 | 9.81% |
| Kyrgyzstan | 41,613 | 1,411 | 22 | 3.39% |
| Lao People's Democratic Republic | 126,023 | 6,275 | 88 | 4.98% |
| Latvia | 29,586 | 2,242 | 117 | 7.58% |
| Lebanon | 105,075 | 9,345 | 180 | 8.89% |
| Lesotho | 55,384 | 2,302 | 110 | 4.16% |
| Liberia | 40,600 | 1,241 | 26 | 3.06% |
| Libya | 110,025 | 10,284 | 153 | 9.35% |
| Lithuania | 31,948 | 1,928 | 69 | 6.04% |
| Luxembourg | 9,931 | 1,088 | 176 | 10.96% |
| Madagascar | 175,831 | 3,560 | 13 | 2.02% |
| Malawi | 141,860 | 3,384 | 18 | 2.39% |
| Malaysia | 469,029 | 30,970 | 99 | 6.60% |
| Maldives | 4,750 | 201 | 40 | 4.24% |
| Mali | 148,178 | 2,994 | 14 | 2.02% |
| Malta | 10,185 | 1,263 | 288 | 12.40% |
| Marshall Islands | 2,432 | 383 | 674 | 15.75% |
| Mauritania | 27,558 | 533 | 13 | 1.94% |
| Mauritius | 85,939 | 13,033 | 1,021 | 15.17% |
| Mexico | 3,884,327 | 404,346 | 324 | 10.41% |
| Micronesia (Federated States of) | 5,494 | 595 | 583 | 10.83% |
| Monaco | 697 | 69 | 185 | 9.94% |
| Mongolia | 19,224 | 480 | 14 | 2.50% |
| Montenegro | 18,897 | 2,067 | 333 | 10.94% |
| Morocco | 683,637 | 51,876 | 144 | 7.59% |
| Mozambique | 267,341 | 5,811 | 20 | 2.17% |
| Myanmar | 1,291,482 | 78,763 | 144 | 6.10% |
| Namibia | 33,619 | 1,209 | 50 | 3.60% |
| Nauru | 346 | 26 | 246 | 7.48% |
| Nepal | 314,535 | 16,599 | 55 | 5.28% |
| Netherlands | 239,129 | 17,562 | 102 | 7.34% |
| New Zealand | 52,078 | 3,207 | 71 | 6.16% |
| Nicaragua | 129,832 | 8,948 | 137 | 6.89% |
| Niger | 109,655 | 1,423 | 6 | 1.30% |
| Nigeria | 1,157,610 | 18,753 | 9 | 1.62% |
| North Macedonia | 79,255 | 9,335 | 434 | 11.78% |
| Norway | 76,384 | 7,040 | 132 | 9.22% |
| Oman | 46,070 | 2,411 | 53 | 5.23% |
| Pakistan | 3,120,862 | 142,112 | 63 | 4.55% |
| Palau | 1,278 | 222 | 1,231 | 17.35% |
| Palestine | 69,309 | 3,817 | 77 | 5.51% |
| Panama | 77,757 | 6,676 | 160 | 8.59% |
| Papua New Guinea | 240,392 | 18,489 | 187 | 7.69% |
| Paraguay | 115,842 | 5,953 | 86 | 5.14% |
| Peru | 306,475 | 12,545 | 37 | 4.09% |
| Philippines | 1,874,860 | 72,508 | 65 | 3.87% |
| Poland | 964,020 | 99,586 | 259 | 10.33% |
| Portugal | 270,198 | 36,191 | 340 | 13.39% |
| Qatar | 35,544 | 3,820 | 133 | 10.75% |
| Republic of Korea | 837,924 | 72,525 | 136 | 8.66% |
| Republic of Moldova | 52,688 | 3,859 | 105 | 7.32% |
| Romania | 317,475 | 24,712 | 128 | 7.78% |
| Russian Federation | 1,898,090 | 98,318 | 67 | 5.18% |
| Rwanda | 94,692 | 2,008 | 16 | 2.12% |
| Saint Kitts and Nevis | 2,038 | 220 | 370 | 10.80% |
| Saint Lucia | 6,770 | 978 | 560 | 14.45% |
| Saint Vincent and the Grenadines | 4,895 | 630 | 557 | 12.87% |
| Samoa | 6,512 | 619 | 293 | 9.50% |
| San Marino | 481 | 43 | 129 | 8.91% |
| Sao Tome and Principe | 1,655 | 51 | 25 | 3.08% |
| Saudi Arabia | 446,934 | 37,208 | 104 | 8.33% |
| Senegal | 157,458 | 5,619 | 37 | 3.57% |
| Serbia | 322,029 | 37,973 | 434 | 11.79% |
| Seychelles | 2,791 | 320 | 314 | 11.48% |
| Sierra Leone | 50,954 | 813 | 10 | 1.60% |
| Singapore | 64,528 | 5,742 | 101 | 8.90% |
| Slovakia | 96,467 | 7,982 | 147 | 8.27% |
| Slovenia | 35,564 | 3,310 | 160 | 9.31% |
| Solomon Islands | 23,687 | 1,801 | 275 | 7.61% |
| Somalia | 166,968 | 3,160 | 16 | 1.89% |
| South Africa | 1,177,378 | 67,290 | 121 | 5.72% |
| South Sudan | 62,141 | 1,263 | 14 | 2.03% |
| Spain | 893,136 | 101,208 | 220 | 11.33% |
| Sri Lanka | 682,129 | 78,530 | 359 | 11.51% |
| Sudan | 357,498 | 17,100 | 42 | 4.78% |
| Suriname | 18,594 | 2,225 | 386 | 11.97% |
| Sweden | 155,694 | 13,646 | 133 | 8.76% |
| Switzerland | 120,052 | 10,697 | 122 | 8.91% |
| Syrian Arab Republic | 235,700 | 17,392 | 120 | 7.38% |
| Taiwan (Province of China) | 585,438 | 51,588 | 218 | 8.81% |
| Tajikistan | 118,361 | 5,425 | 57 | 4.58% |
| Thailand | 1,468,667 | 112,052 | 160 | 7.63% |
| Timor-Leste | 16,474 | 621 | 47 | 3.77% |
| Togo | 54,176 | 1,011 | 13 | 1.87% |
| Tonga | 3,408 | 319 | 312 | 9.36% |
| Trinidad and Tobago | 75,221 | 11,250 | 811 | 14.96% |
| Tunisia | 230,018 | 23,723 | 205 | 10.31% |
| Turkey | 1,301,538 | 88,537 | 109 | 6.80% |
| Turkmenistan | 70,689 | 3,607 | 71 | 5.10% |
| Tuvalu | 549 | 63 | 533 | 11.46% |
| Uganda | 279,067 | 6,185 | 15 | 2.22% |
| Ukraine | 501,310 | 29,487 | 67 | 5.88% |
| United Arab Emirates | 139,077 | 12,742 | 138 | 9.16% |
| United Kingdom | 1,237,737 | 165,207 | 246 | 13.35% |
| United Republic of Tanzania | 390,470 | 7,431 | 13 | 1.90% |
| United States of America | 8,129,016 | 1,011,874 | 309 | 12.45% |
| Uruguay | 50,630 | 3,030 | 88 | 5.98% |
| Uzbekistan | 587,342 | 29,415 | 87 | 5.01% |
| Vanuatu | 8,056 | 651 | 221 | 8.08% |
| Venezuela (Bolivarian Republic of) | 680,603 | 58,565 | 209 | 8.60% |
| Viet Nam | 1,770,069 | 92,708 | 96 | 5.24% |
| Yemen | 204,341 | 6,242 | 20 | 3.05% |
| Zambia | 142,671 | 3,022 | 17 | 2.12% |
| Zimbabwe | 206,599 | 7,849 | 52 | 3.80% |

Table B. Countries in each Global Burden of Disease Super Region

| **Super Region of GBD Project** | **Country** |
| --- | --- |
| **Central Europe, Eastern Europe, and Central Asia** | Albania |
|  | Armenia |
|  | Azerbaijan |
|  | Belarus |
|  | Bosnia and Herzegovina |
|  | Bulgaria |
|  | Croatia |
|  | Czechia |
|  | Estonia |
|  | Georgia |
|  | Hungary |
|  | Kazakhstan |
|  | Kyrgyzstan |
|  | Latvia |
|  | Lithuania |
|  | Mongolia |
|  | Montenegro |
|  | North Macedonia |
|  | Poland |
|  | Republic of Moldova |
|  | Romania |
|  | Russian Federation |
|  | Serbia |
|  | Slovakia |
|  | Slovenia |
|  | Tajikistan |
|  | Turkmenistan |
|  | Ukraine |
|  | Uzbekistan |
| **High-income** | Andorra |
|  | Argentina |
|  | Australia |
|  | Austria |
|  | Belgium |
|  | Brunei Darussalam |
|  | Canada |
|  | Chile |
|  | Cyprus |
|  | Denmark |
|  | Finland |
|  | France |
|  | Germany |
|  | Greece |
|  | Greenland |
|  | Iceland |
|  | Ireland |
|  | Israel |
|  | Italy |
|  | Japan |
|  | Luxembourg |
|  | Malta |
|  | Monaco |
|  | Netherlands |
|  | New Zealand |
|  | Norway |
|  | Portugal |
|  | Republic of Korea |
|  | San Marino |
|  | Singapore |
|  | Spain |
|  | Sweden |
|  | Switzerland |
|  | United Kingdom |
|  | United States of America |
|  | Uruguay |
| **Latin America and Caribbean** | Antigua and Barbuda |
|  | Bahamas |
|  | Barbados |
|  | Belize |
|  | Bermuda |
|  | Bolivia (Plurinational State of) |
|  | Brazil |
|  | Colombia |
|  | Costa Rica |
|  | Cuba |
|  | Dominica |
|  | Dominican Republic |
|  | Ecuador |
|  | El Salvador |
|  | Grenada |
|  | Guatemala |
|  | Guyana |
|  | Haiti |
|  | Honduras |
|  | Jamaica |
|  | Mexico |
|  | Nicaragua |
|  | Panama |
|  | Paraguay |
|  | Peru |
|  | Puerto Rico |
|  | Saint Kitts and Nevis |
|  | Saint Lucia |
|  | Saint Vincent and the Grenadines |
|  | Suriname |
|  | Trinidad and Tobago |
|  | United States Virgin Islands |
|  | Venezuela (Bolivarian Republic of) |
| **North Africa and Middle East** | Afghanistan |
|  | Algeria |
|  | Bahrain |
|  | Egypt |
|  | Iran (Islamic Republic of) |
|  | Iraq |
|  | Jordan |
|  | Kuwait |
|  | Lebanon |
|  | Libya |
|  | Morocco |
|  | Oman |
|  | Palestine |
|  | Qatar |
|  | Saudi Arabia |
|  | Sudan |
|  | Syrian Arab Republic |
|  | Tunisia |
|  | Turkey |
|  | United Arab Emirates |
|  | Yemen |
| **South Asia** | Bangladesh |
|  | Bhutan |
|  | India |
|  | Nepal |
|  | Pakistan |
| **Southeast Asia, East Asia, and Oceania** | American Samoa |
|  | Cambodia |
|  | China |
|  | Cook Islands |
|  | Democratic People's Republic of Korea |
|  | Fiji |
|  | Guam |
|  | Indonesia |
|  | Kiribati |
|  | Lao People's Democratic Republic |
|  | Malaysia |
|  | Maldives |
|  | Marshall Islands |
|  | Mauritius |
|  | Micronesia (Federated States of) |
|  | Myanmar |
|  | Nauru |
|  | Niue |
|  | Northern Mariana Islands |
|  | Palau |
|  | Papua New Guinea |
|  | Philippines |
|  | Samoa |
|  | Seychelles |
|  | Solomon Islands |
|  | Sri Lanka |
|  | Taiwan (Province of China) |
|  | Thailand |
|  | Timor-Leste |
|  | Tokelau |
|  | Tonga |
|  | Tuvalu |
|  | Vanuatu |
|  | Viet Nam |
| **Sub-Saharan Africa** | Angola |
|  | Benin |
|  | Botswana |
|  | Burkina Faso |
|  | Burundi |
|  | Côte d'Ivoire |
|  | Cabo Verde |
|  | Cameroon |
|  | Central African Republic |
|  | Chad |
|  | Comoros |
|  | Congo |
|  | Democratic Republic of the Congo |
|  | Djibouti |
|  | Equatorial Guinea |
|  | Eritrea |
|  | Eswatini |
|  | Ethiopia |
|  | Gabon |
|  | Gambia |
|  | Ghana |
|  | Guinea |
|  | Guinea-Bissau |
|  | Kenya |
|  | Lesotho |
|  | Liberia |
|  | Madagascar |
|  | Malawi |
|  | Mali |
|  | Mauritania |
|  | Mozambique |
|  | Namibia |
|  | Niger |
|  | Nigeria |
|  | Rwanda |
|  | Sao Tome and Principe |
|  | Senegal |
|  | Sierra Leone |
|  | Somalia |
|  | South Africa |
|  | South Sudan |
|  | Togo |
|  | Uganda |
|  | United Republic of Tanzania |
|  | Zambia |
|  | Zimbabwe |
